# Falling treatment uptake in the hepatitis C care cascade is a growing threat to achieving elimination

**DOI:** 10.1111/jvh.13757

**Published:** 2022-11-02

**Authors:** Kathleen Bryce, Colette Smith, Alison Rodger, Douglas Macdonald

**Affiliations:** ^1^ Institute for Global Health University College London London UK; ^2^ Royal Free London NHS Foundation Trust London UK; ^3^ Institute for Liver and Digestive Health University College London London UK

**Keywords:** antiviral agents, hepatitis C, risk factors, substance abuse, intravenous, time‐to‐treatment

## Abstract

Most high‐income countries are not on track to achieve the World Health Organization hepatitis C elimination targets. As elimination programmes assess growing proportions of patients in community‐based pathways, rates of treatment uptake may fall. We aimed to identify factors associated with DAA treatment uptake and measure changes in their prevalence over time. We performed a time‐to‐treatment analysis on 2728 patients approved for hepatitis C Direct‐Acting Antiviral treatment in the North Central London region between January 2016 and October 2019. We investigated the association between treatment uptake and factors including assessment/treatment setting (hospital, drug service or prison), patient age, gender, injection drug use, harmful alcohol use, cirrhosis status and previous treatment. The likelihood of treatment uptake was reduced by three independent risk factors. These included assessment setting: prison‐based or drug‐service pathways (aHR 0.29 or 0.81 vs. hospital outpatient pathway, 95% CI 0.21–0.40 and 0.70–0.94 respectively, *p* < .001); being UK‐born (aHR 0.89 vs. non‐UK born, 0.82–0.98, *p* = .01); and history of harmful alcohol use (aHR 0.84 vs. no history, 0.72–0.99, *p* = .04). The average number of these risk factors for not starting treatment per patient increased over time (*R* ^2^ = 0.66 *p* < .001). Independent of these, there was an additional 5% reduction in rate of treatment initiation in each successive year of the programme (aHR 0.95, 0.91–0.99, *p* = .02). In conclusion, disengagement from care before treatment uptake was found to be a growing threat to elimination. Despite provision of community‐based test‐to‐cure pathways, there are persistent barriers to treatment uptake and these are increasing over time.

**Abbreviations**
DAA: direct‐acting antiviral
HCV: hepatitis C virus
IDU: injection drug use
MDT: multidisciplinary team
NCL: north central London
ODN: operational delivery network
PMS: patient management system
PWID: people who inject drugs
WHO: World Health Organization

## 1 INTRODUCTION

The World Health Organization (WHO) has set a goal for the elimination of hepatitis C as a public health threat by 2030, defined as an 90% reduction in incidence and 65% reduction in hepatitis C‐attributable death.^1^ In the absence of a vaccine, this must be achieved through treatment with highly‐effective direct‐acting antivirals (DAAs),^2^ though it is widely acknowledged that enhanced case‐finding and transmission‐reduction measures will also be necessary.^1^, ^3^, ^4^ There are approximately 118,000 people with hepatitis C virus (HCV) infection in the UK (2019) and the majority of these are people who inject drugs (PWID).^5^, ^6^ Despite widely‐available DAA therapy, there is no evidence of a fall in HCV incidence in PWID since 2015^7^, ^8^ and only 38% of people with hepatitis C in the UK are estimated to have been successfully treated (2019).^5^

Dynamic mathematical transmission modelling using global data from 2015 to 2016 DAA elimination programmes^9^ reported that the WHO target may be achieved by 2032. This model and others^6^, ^10^ assume that the rates of treatment uptake in those diagnosed will remain constant in all programmes from the introduction of DAAs onwards. However, whilst DAA therapy has improved treatment uptake compared with the Interferon‐based treatment era,^5^, ^10^, ^11^ uptake remains low in some important groups, particularly PWID. In Canada, DAA treatment uptake was reported in 45% of current injectors (2018)^12^ and an Australian study observed that 34% of PWID with hepatitis C started DAA treatment between 2014 and 2018.^11^ In the US, treatment uptake amongst people experiencing homelessness was a rate‐limiting step along the care cascade, with 5%–59% of those chronically infected starting DAAs.^13^ A recent meta‐analysis of 20 population‐based studies reporting the HCV care cascade (published 2017–2020)^14^ found DAA uptake in PWID ranged between 13% in the US, 37% in Australia, 40% in Canada and 50% in Georgia.

DAA treatment uptake rates are falling in high income countries and 76% of these are not on track to achieve elimination targets, according to updated analyses.^15^, ^16^ Falling treatment uptake has been highlighted as a risk to elimination even in Australia, an early adopter of universal unrestricted DAA access.^17^ This emphasizes that strategies for maintaining patient engagement in care from diagnosis to treatment will be critical to elimination.

Patient groups with high rates of disengagement between treatment approval and initiation are likely to account for an increasing proportion of untreated cohorts as elimination programmes move from hospital to community‐based pathways. Even if total numbers of patients entering the care cascade are increased by case‐finding initiatives, it is unclear whether treatment uptake rates can be sustained at sufficient levels for elimination. Additionally, current modelling estimates do not account for COVID‐19‐related disruptions in HCV care,^15^ estimated to cause significant excess mortality over the next decade.^18^

We aimed to identify factors associated with treatment uptake and investigate whether the risk factors for not starting treatment were becoming more prevalent over time. We also aimed to determine whether rates of treatment uptake after approval are changing as the hepatitis C elimination programme progresses in North Central London.

## 2 METHODS

We analysed a cohort of 2728 patients assessed and approved for DAA treatment in the North Central London (NCL) hepatitis C operational delivery network (ODN) between January 2016 and October 2019. In addition to 8 partner hospital sites, the NCL ODN delivers embedded community‐only hepatitis C diagnosis‐to‐cure pathways in 14 drug and alcohol services and two prisons, one of which is a large remand prison with a turnover of approximately 300 inmates per month. In each community service, a clinical nurse specialist provides assessment (blood tests, Fibroscan®), treatment counselling and initiation, treatment monitoring and outcome testing, such that the patient does not have to visit services outside the one with which they are already engaged. Consultations are aligned to opioid substitution therapy supervision in drug and alcohol services and hospital investigations are only required in those with cirrhosis who require surveillance for liver cancer and/or varices. The ODN also includes two large HIV services in which hepatitis C treatment is aligned to HIV care.

Data were routinely prospectively collected in all patients through a bespoke patient management system (PMS) which time‐stamps progress through stages of the care cascade and pathway processes. This permits measurement of duration of “system‐only” stages, such as drug–drug interaction checking, application for high‐cost drug approval, prescribing, dispensing and couriering treatment courses to community‐based centres. NHS England mandated a clinical prioritization scheme until 2018^19^ and the NCL PMS included a clinical scoring system based on objective parameters that listed patients for multidisciplinary team (MDT) discussion and approval once a threshold score was met. This ensures that a time‐to‐event analysis between MDT approval and treatment was not directly influenced by clinical priority.

The primary outcome was initiation of hepatitis C treatment and a time‐to‐treatment analysis was performed. The sample size was constrained by available data but it was adequate to allow detection of a hazard ratio of 0.84 for factors that occur in at least 10% of study participants (80% power and 5% Type I error). Patient‐related variables in the model included age, gender, a history of intravenous drug use (IDU), born in the UK or elsewhere, interferon treatment experience, hepatocellular carcinoma, a significant alcohol history felt to be contributing to liver disease, liver transplantation, need for renal dialysis, HIV status, viral HCV genotype and liver disease stage. Pathway‐related factors included assessment and treatment setting (hospital outpatients, drug and alcohol service or prison) and when in the DAA programme the patient was referred for treatment (months 1–46, starting from January 2016).

Previous studies investigating treatment uptake rates in the DAA era have used a multivariable logistic regression analysis to identify factors associated with this outcome.^11^, ^20^, ^21^, ^22^ This is problematic because treatment is not necessarily permanently declined if not initiated within a study period. This approach may also be confounded by lead‐time bias because those enrolled at the beginning of the study interval have a longer opportunity to commence treatment than those closer to the end. Furthermore, logistic analysis loses information about duration of delay and the trajectory of progression to treatment in each group with time. We therefore used a multivariable Cox regression time‐to‐event analysis to identify factors associated with initiating treatment from the time origin of treatment approval, after ensuring that the proportional hazards assumptions were met. For those not starting treatment, administrative censoring occurred on the date of study database closure, at least 6 months after the last recruited patient was approved for treatment (an interval which is longer than the mean approval‐to‐treatment time). The factors included in the adjusted analysis were selected based on statistical significance at the 5% level, as well as biological plausibility, the potential to be mediators and previously identified confounders. There were no missing values in the dataset.

To evaluate the impact of co‐existent factors which negatively impact on likelihood of treatment uptake, we performed a secondary time‐to‐treatment analysis considering the number of risk factors for not starting treatment that an individual had.

Elimination programmes will accumulate patients whose progression from approval to treatment is delayed, such that the average duration of delay will inevitably increase. This confounds estimates of whether delays between approval and treatment are increasing as the elimination programme continues. We therefore performed a correlation analysis between the month of assessment (from beginning of the programme) and the mean number of risk factors associated with not starting treatment in all patients presenting in that month. The dependent variable (mean number of risk factors) was normally distributed so a Pearson's correlation coefficient was derived.

To investigate the impact of delays between treatment approval and initiation on healthcare team workload, data were also collected on the number of contacts made with patients by pathway navigators and nurse specialists between the treatment approval and start dates. A correlation analysis was performed between approval‐to‐treatment time and number of healthcare contacts recorded, deriving a Spearman's rank correlation coefficient due to the non‐parametric distribution of the dependent variable (number of contacts).

Ethical approval was obtained from the Health Research Authority (REC reference 21/HRA/0929). Informed consent was not required to analyse routinely collected pseudonymized data. All analyses were performed in SPPS version 26 (IBM corporation).

## 3 RESULTS

### 3.1 Study cohort characteristics

The baseline characteristics of the patient cohort are shown in Table 1. Most patients were male (69%), born in the UK (70%) and treated in hospital outpatients (83%). 14% were treated in a drug and alcohol service and 3% in prison. 41% of patients had a history of injection drug use (IDU) and in 7% alcohol was deemed to be a contributor to liver disease at baseline. Table 2 shows that the proportion of patients treated in non‐hospital settings, with a history of IDU or harmful alcohol use and born in the UK increasing significantly over time. The proportion with cirrhosis did not differ between 2016 and 2019.

**TABLE 1:** Cohort characteristics

		Treatment uptake, *n* (%)
Age (years)		
Mean (SD; range)	50 (12.5; 18–92)	
Gender		
Male	1888 (69%)	1668 (88%)
Female	840 (31%)	774 (92%)
Pathway setting		
Hospital outpatients	2272 (83%)	2114 (93%)
Drug and alcohol service	377 (14%)	290 (77%)
Prison	79 (3%)	38 (48%)
Injection drug use (ever)		
Yes	1130 (41%)	956 (85%)
No	1598 (59%)	1486 (93%)
Born in UK		
Yes	1914 (70%)	1686 (88%)
No	814 (30%)	756 (93%)
Region of birth, if not UK		
Other European	483 (17%)	445 (92%)
Eastern Mediterranean	144 (5%)	136 (94%)
Africa	84 (3%)	77 (92%)
Americas	44 (2%)	41 (93%)
Western Pacific	38 (1%)	37 (97%)
Southeast Asia	21 (1%)	19 (90%)
Previous treatment		
Yes	496 (18%)	454 (92%)
No	2232 (82%)	1988 (89%)
Hepatocellular carcinoma		
Yes	27 (1%)	19 (70%)
No	2701 (99%)	2423 (90%)
Alcohol contributing to liver disease		
Yes	201 (7%)	164 (82%)
No	2527 (93%)	2278 (90%)
Liver transplant		
Yes	43 (2%)	42 (98%)
No	2685 (98%)	2400 (89%)
Renal dialysis		
Yes	19 (1%)	16 (84%)
No	2709 (99%)	2426 (90%)
HIV		
Yes	371 (14%)	348 (94%)
No	2357 (86%)	2094 (89%)
HCV genotype		
1	1598 (59%)	1457 (91%)
2	141 (5%)	125 (89%)
3	711 (26%)	598 (84%)
4	231 (8%)	217 (94%)
5, 6	17(1%)	15 (88%)
Other	30 (1%)	30 (100%)
Cirrhosis status		
Compensated	449 (16%)	408 (91%)
Decompensated	55 (2%)	45 (82%)
Non‐cirrhotic	2224 (82%)	1989 (89%)
Number of risk factors		
0	695 (25%)	664 (96%)
1	1556 (57%)	1420 (91%)
2 or more	477 (17%)	358 (75%)
Total	2728	

Abbreviation: SD, standard deviation.

**TABLE 2:** Cohort characteristics over time

Year approved	2016	2019	*p*
No. (%)			
Male	529 (68%)	272 (75%)	.01
Non‐hospital pathway (drug and alcohol service or prison)	52 (7%)	133 (37%)	<.001
Injection drug use (ever)	235 (30%)	215 (59%)	<.001
Born in UK	516 (66%)	266 (73%)	.01
Alcohol contributing to liver disease	39 (5%)	39 (11%)	<.001
Cirrhosis	161 (21%)	75 (21%)	.98
Total	780	362	

2442 patients (90%) commenced treatment between January 2016 and October 2019 and the median time from approval to starting treatment or censor was 51 days (range 0–1509). Delays between MDT approval and treatment which were attributable to system‐only stages in the pathway (e.g., pharmacy checks, dispensing) across all treatment settings (data available in 1611 cases) were on average 3.7 days duration (95% CI 3.3–4.2), representing only 8% of the median time between MDT approval and treatment in this group.

### 3.2 Factors associated with DAA treatment uptake

Variables associated with the likelihood of treatment initiation after approval are shown in Table 3. Seven different factors were shown to be independently associated with treatment in an adjusted analysis. There was a significant decline in likelihood of DAA treatment uptake by each year of the programme in which patients were referred: a successive 5% reduction for each 12‐month period from the beginning of the DAA programme, independent of all other factors included in the model.

**TABLE 3:** Factors associated with progression to HCV treatment uptake

Variable	Unadjusted			Adjusted		
	HR	95% CI	*p*	aHR	95% CI	*p*
Age (per 10 years)	1.01	0.98, 1.04	.66	0.97	0.94, 1.01	.09
Male gender (vs. female)	0.93	0.86, 1.01	.10	0.99	0.91, 1.09	.88
Pathway setting						
Prison	0.27	0.19, 0.37	<.001	0.29	0.21, 0.40	<.001
Drug and alcohol service	0.71	0.63, 0.81		0.81	0.70, 0.94	
Hospital outpatients	REF			REF		
Injection drug use (ever) (yes vs. no)	0.74	0.69, 0.81	<.001	0.92	0.83, 1.01	.09
Born in UK (Yes vs. No)	0.85	0.78, 0.92	<.001	0.89	0.82, 0.98	.01
Previous treatment (yes vs. no)	1.05	0.95, 1.16	.35	0.97	0.88, 1.08	.62
Hepatocellular carcinoma (yes vs. no)	0.64	0.41, 1.00	.05	0.70	0.44, 1.11	.13
Alcohol contributing to liver disease (yes vs. no)	0.77	0.65, 0.90	.001	0.84	0.72, 0.99	.04
Liver transplant (yes vs. no)	1.20	0.88, 1.62	.25	1.16	0.85, 1.58	.35
Renal dialysis (yes vs. no)	0.65	0.40, 1.06	.08	0.60	0.37, 0.98	.04
HIV (Positive vs. Negative)	1.37	1.23, 1.54	<.001	1.23	1.09, 1.39	.001
HCV genotype						
Genotype 2, 3, 5, 6 or ‘other’	0.78	0.72, 0.85	<.001	0.83	0.76, 0.91	<.001
Genotype 1 or 4	REF			REF		
Cirrhosis status						
Compensated cirrhosis	1.04	0.93, 1.16	.19	1.12	1.00, 1.25	.07
Decompensated cirrhosis	0.79	0.59, 1.05		0.85	0.63, 1.15	
Non‐cirrhotic	REF					
Month of DAA programme at referral (per 12 months)	0.90	0.87, 0.94	<.001	0.95	0.91, 0.99	.02

*Note*: Cox regression analysis showing unadjusted and adjusted impact of variables on DAA treatment initiation after MDT approval.

Abbreviation: REF, reference value.

The setting of the assessment and treatment pathway had the most significant impact on likelihood of treatment in the Cox regression analysis. Patients assessed in prison were 70% less likely to start treatment compared with patients assessed in hospital clinic settings (aHR 0.29, 95% CI 0.21–0.40, *p* < .001). Assessment and treatment in drug and alcohol services was associated with a 19% lower likelihood of commencing treatment, compared with hospital (aHR 0.81, CI 0.70–0.94, *p* < .001), despite the provision of in‐house hepatitis C assessment and treatment monitoring aligned to opioid substitution therapy. A history of IDU was only associated with a reduced likelihood of commencing treatment in unadjusted analysis and became non‐significant in adjusted analysis, suggesting a correlation and more meaningful effect of drug‐service setting than IDU. However, a determination by the referring clinician that alcohol was a significant contributor to liver disease was independently associated with lower likelihood of starting treatment in the adjusted model (aHR 0.84, CI 0.72–0.99, *p* = .04). This association became non‐significant when prisons‐based pathway settings were excluded from analysis (Supporting information).

Those of UK origin were less likely to start treatment than those born outside the UK (aHR 0.89, CI 0.82–0.98, *p* = .01). Age, gender, previous treatment, liver disease stage, hepatocellular carcinoma and liver transplantation were not independently associated with likelihood of progression to treatment, although the numbers in the analysis were low for the latter two factors.

There was reduced treatment uptake in patients requiring dialysis (aHR 0.60, CI 0.37–0.98, *p* = .04) and those infected with genotypes other than 1 or 4 (aHR 0.38, CI 0.76–0.91, *p* < .001), which is attributable to patients meeting clinical priority thresholds and MDT approval in advance of NHS commissioning of dialysis‐compatible and genotype 3 treatments, which occurred during the study interval. HIV co‐infection was a strong positive predictor of treatment uptake (aHR 1.23, CI 1.09–1.39, *p* = .001).

### 3.3 Co‐existent risk factors

Based on the findings from the primary analysis, a secondary time‐to‐treatment Cox regression analysis compared patients grouped by their number of co‐existent risk factors:

1. Pathway setting: assessment for treatment in prison or in drug and alcohol service
2. Born in the UK
3. Harmful alcohol use.

**FIGURE 1:**
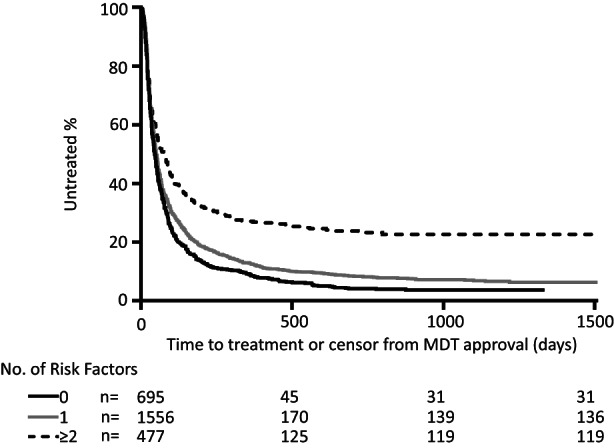
Progression to treatment uptake (or censor) from multidisciplinary team (MDT) approval by number of risk factors for disengagement. The numbers of patients in each group at 500, 1000 and 1500 days are shown in the underlying table. Those who had two or more risk factors (*n* = 477) had a 42% lower likelihood of treatment than those with no risk factors (*n* = 695) (HR 0.58, CI 0.51–0.65, *p* < .001, Cox regression).

Renal dialysis and genotype were not included because the prolonged duration between approval and treatment were attributable to historical changes in treatment availability, as previously described. The month of referral into programme was also excluded as a time‐dependent variable which would confound the trajectory of progress to treatment in the group over time. Increasing numbers of any of these three risk factors decreased the likelihood of starting treatment (Figure 1). Those with two or more risk factors (*n* = 477) had a 42% lower rate of treatment than those with no risk factors (*n* = 695) (HR 0.58, CI 0.51–0.65, *p* < .001). Importantly, the Kaplan–Meier curve of this group plateaus, indicating a ceiling of treatment uptake in those with multiple risk factors for disengagement before treatment uptake.

**FIGURE 2:**
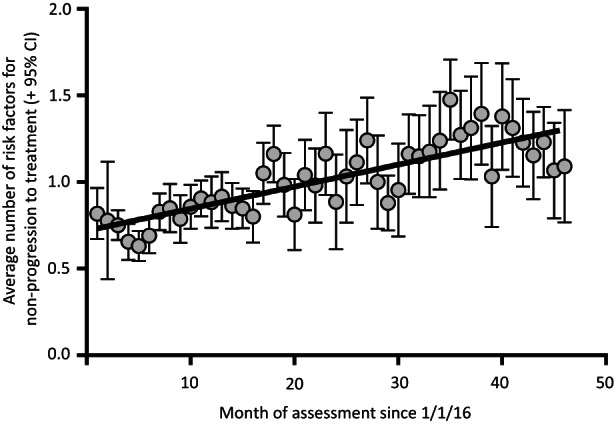
Average prevalence of risk factors for disengagement before treatment by month of assessment. The average number of risk factors (+95% CI) that negatively impacted progression to treatment uptake (excluding month of programme, dialysis and genotype) increased by month of assessment since the beginning of the direct‐acting antiviral elimination programme (Pearson *R* ^2^ = 0.66, *p* < .0001).

Duration of time between approval and treatment increased as the programme progressed (Supporting information) and this may be related to changes over time in the risk factor profile of patients. However, this is affected by lead‐time bias since those treated more recently had a longer potential interval from approval. We therefore examined whether the three independent risk factors for not starting treatment outlined above were becoming more prevalent. Figure 2 shows a strong correlation between the average number of these risk factors per patient and month of assessment in the DAA programme (Pearson *R* ^2^ = 0.66, *p* < .0001). Assessment in pathways embedded in drug and alcohol services is increasing in the NCL ODN region (5% of those approved for treatment in 2016; 34% in 2019, *p* < .001), as is the proportion of those with a history of IDU (30% in 2016 to 59% in 2019, *p* < .001) and the proportion of patients in whom alcohol is a significant contributor to liver disease (5% in 2016 to 11% in 2019, *p* < .001).

### 3.4 Impact on healthcare team workload

**FIGURE 3:**
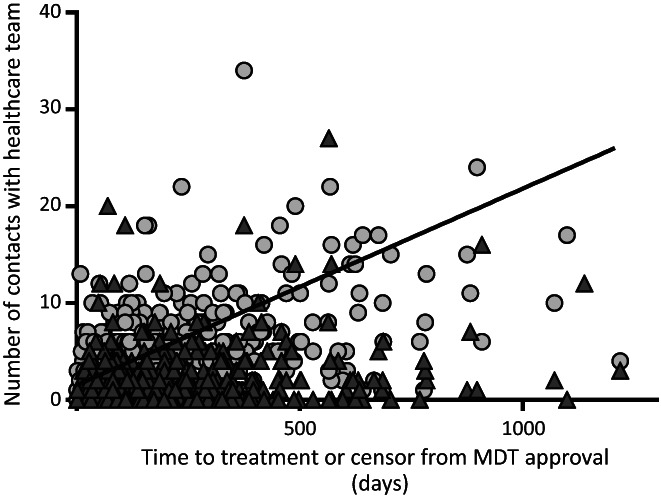
Healthcare system workload increases with delay between approval and treatment. Healthcare workload shown as number of recorded contacts between nurse specialists (triangle) and navigators (circle) and time between approval and treatment. Linear regression line shown is for all contacts, Spearman *R* = 0.6, *p* < .0001).

We examined the relationship between the number of patient contacts made by NCL ODN staff (pathway navigators and clinical nurse specialists) and the time between MDT approval and treatment initiation (or censor, Figure 3). This showed a significant correlation between delay at this stage of the care cascade and the number of contacts with the healthcare team required to progress to treatment (Spearman *R* = 0.6, *p* < .0001).

## 4 DISCUSSION

This analysis of a large cohort of patients treated across a range of settings in North Central London has identified three independent risk factors for patient disengagement before hepatitis C DAA treatment uptake: assessment for treatment in prison or in drug and alcohol services reduced the likelihood of treatment uptake by 70% or by 19%, respectively; harmful alcohol use reduced treatment uptake by 16% and being born in the UK reduced treatment uptake by 11%. A combination of these risk factors conferred a higher risk of not starting treatment after approval—patients with two or more of these risk factors had a 42% lower rate of treatment initiation than those with no risk factors—and their coexistence was becoming increasingly prevalent. Another important finding was that the likelihood of starting treatment declined over time (5% reduction in likelihood of treatment uptake per year), independently of these risk factors. Not being engaged with hepatitis C treatment increases the risks of onward transmission, individual harm and healthcare workload and is a growing threat to hepatitis C elimination.

Enhancing case‐finding and treatment uptake are both essential for achieving HCV elimination. Whilst diagnosis rates are relatively easy to measure, determining whether delays between stages in the care cascade are increasing is hampered by study interval biases and the lack of national‐level data encompassing accurately time‐stamped progress through the care cascade. We have analysed a cohort in which these data were available and have identified two separate phenomena which indicate that patient disengagement before treatment uptake is increasing as our regional elimination programme continues. The first is that there are several independent risk factors for disengagement and their prevalence in individual patients is rising as the DAA programme progresses. The second phenomenon is that the likelihood of starting treatment is reducing over time, independently of these risk factors and their growing prevalence. One explanation for this may be the “warehouse effect”.^23^, ^24^, ^25^ Breakthrough treatments will inevitably be given first to “queued” patients who have sustained engagement prior to the treatment becoming available. The observed reduction in likelihood of treatment uptake in patients presenting subsequently (and the increase in the number of healthcare contacts needed to initiate treatment) may simply represent transition from a well‐engaged population towards a rate of treatment uptake more representative of the whole population of patients with hepatitis C who experience many barriers to healthcare.

Together, these undermine the key assumption made in models of trajectories towards elimination that treatment uptake rates at the beginning of elimination programmes will be maintained.^6^, ^9^, ^10^ Although overall non‐progression to treatment in this cohort is low (10%), we have examined only one segment of the HCV care cascade, from MDT treatment approval to treatment initiation. Nonetheless, projection from our linear regression analysis suggests that if current trends continue then by 2023 the average patient approved for treatment will have two or more risk factors for disengagement before treatment uptake, which is currently associated with a rate of non‐progression to treatment of 30%. Importantly, our findings predate the COVID‐19‐related service disruptions and their negative impact on testing and treatment rates.^18^, ^26^, ^27^, ^28^

Some of the factors impacting treatment uptake identified herein have been reported previously. Difficulties in initiating treatment in prison settings have been recognized in the literature.^29^, ^30^ The majority of patients in prison pathways in our network are on remand with short and unpredictable periods of incarceration and release, which can complicate treatment initiation and completion, consistent with experience elsewhere in the UK.^31^

People who inject drugs experience many barriers to hepatitis C treatment,^32^ although this population achieve cure rates similar to other groups once engaged.^33^ Recent studies show that there is still significant uncertainty about DAA treatment amongst those affected.^34^, ^35^ Stigma and negative experiences of the healthcare system continue to act as barriers for this population.^36^ An interventional study in the UK (HepCATT) found barriers to engagement in drug services including lack of hepatitis C knowledge, fear of diagnosis and treatment, unstable living circumstances and service‐related barriers; however, these could be overcome with personalized care and peer support.^37^, ^38^ It is noteworthy that the NCL programme has yet to engage significant numbers of people actively injecting drugs in needle‐exchange programmes and treatment uptake in these groups is typically lower than those engaged in opioid substitution programmes.^21^, ^39^, ^40^ A UK study is underway to examine if pathways embedded in needle exchange pharmacy services maintain engagement.^41^

Harmful alcohol use (as determined by assessing clinician) was associated with reduced treatment uptake in NCL and alcohol use disorders have been identified in the literature as a barrier to DAA treatment uptake in cohort studies in Canada^42^, ^43^ and France.^44^ Encouragingly, pharmacological treatment for alcohol use disorder removed this association in one study.^44^

The finding that those born outside the UK were more likely to start DAA treatment may be explained by our analysis not adjusting for differences in socioeconomic status (the so‐called ‘healthy immigrant’ effect^45^). It is also possible that migrant populations in NCL did experience barriers to access at earlier stages of the care cascade (such as testing or linkage to care) but that the study design did not allow measurement of these. Relatively little is known about the barriers to hepatitis C treatment uptake in migrants to the UK. Barriers to hepatitis C testing in this group include lack of registration with primary care and inadequate screening practices,^46^ language barriers, long working hours and low levels of trust in their general practioner.^47^

The positive association between HIV co‐infection and increased likelihood of treatment uptake (particularly if engaged with antiretroviral therapy^48^) has been observed in other studies in the DAA era.^39^, ^49^, ^50^ HIV services are unlikely to refer patients who are not engaged with care and people living with HIV have an established routine for daily drug therapy. They are supported by additional services in the UK including a parallel programme aiming to eliminate HCV co‐infection in people living with HIV.^23^

Failure to maintain patient engagement in care until treatment initiation is an important and growing threat to hepatitis C elimination. However, if the risks for this can be identified, disengagement before treatment uptake can be prevented. In the NCL network, the results of this study have allowed us to target evidence‐based interventions, namely voucher incentives^51^, ^52^, ^53^ and peer support^54^, ^55^ to those most at risk of disengagement from care before treatment. We have also piloted a same‐day peer‐led treatment protocol which evaluated treatment uptake and adherence when this was commenced on the same day as diagnosis (manuscript in preparation). The setting‐related risk factors identified have also allowed us to address specific pathway‐based failures in ensuring treatment initiation, for example, by providing remand prisoners with an emergency supply of DAAs if they are released.

We propose that the amount of time spent by a patient at each stage of the HCV care cascade is an essential metric that should be routinely collected and reported. This would allow swift identification of changing factors that might impede progression through the care cascade and rapid diversion of resources to address them, at both the local and national level.

We recognize that this study was unable to analyse engagement in earlier stages of the care cascade, such as uptake of testing and referral, due to lack of data from other healthcare organizations where these events take place. Analysis of delays between assessment and MDT approval would have been confounded by thresholds for approval set by the clinical prioritization system that existed prior to 2018. Nonetheless, the growing delays we have observed between approval and treatment are alone sufficient to derail progress to elimination and there is an urgent need to examine whether increases in disengagement are also occurring upstream in the care cascade as the programme progresses. We did not explicitly account for death as a competing risk as only 0.8% (21/2728) of the study population died without treatment, which would not impact significantly upon the analysis. The lack of socioeconomic data (or complete postcode data from which this can be derived) means this important factor has been omitted from our model. We have also used a crude surrogate of healthcare workload in number of patient contacts by NCL ODN staff before treatment, which does not capture the efforts of peer support workers and community services to engage patients. The generalizability of the results is reduced by region‐specific factors, such as having a large remand (rather than long‐stay) prison population; however, the baseline patient characteristics of this cohort are typical of populations with hepatitis C in high‐income countries.

Further qualitative investigation is needed to explore influences on patient engagement with the hepatitis C care cascade in the UK in the DAA era, such as the effects of mental health and socioeconomic status. In future, these risk factors could be combined with dynamic real‐time data on patient interaction with one stage of the pathway, such that disengagement from a subsequent stage can be pre‐empted with supportive interventions. It will also be important to evaluate the impact of these factors on testing and treatment uptake in the post‐COVID‐19 pandemic era, as disruption to health and social care services is likely to have aggravated existing barriers to care.

In conclusion, disengagement from care between treatment approval and initiation was found to be a substantial and growing threat to hepatitis C elimination in London. The identified risk factors for disengagement before treatment uptake are increasing in prevalence and a combination of these significantly reduces the likelihood of treatment initiation. There is an additional independent reduction in treatment uptake with each year of the elimination programme, despite the provision of community‐based pathways. This analysis is of value to those deciding how to distribute resources between stages in the hepatitis care cascade to accelerate progress to elimination and also for targeting support for treatment uptake towards those most in need.

## AUTHOR CONTRIBUTIONS

DM developed the concept for the study and built the patient management system from which data were acquired. DM, KB, CS and AR were involved in designing the study. DM and KB acquired, analysed and interpreted the data with statistical supervision by CS. KB drafted and DM, CS and AR revised the manuscript. All authors read and approved the final manuscript.

## FUNDING INFORMATION

No external financial support was received for the conduct of the research or preparation of the article.

## CONFLICT OF INTEREST

Dr Smith reports grants from ViiV Healthcare and personal fees from Gilead Sciences Ltd, outside the submitted work. The other authors declare no competing interests.

## Supporting information


Data S1

## Data Availability

The data that support the findings of this study are available from the corresponding author upon reasonable request.
