# Supplementary material for: Falling treatment uptake in the hepatitis C care cascade is a growing threat to achieving elimination
Source: J Viral Hepat. 2022 Nov 2;30(1):46–55. doi: 10.1111/jvh.13757 (PMC10091771; doi:10.1111/jvh.13757)

**Supporting information**

**Title: Falling treatment uptake in the hepatitis C care cascade is a growing threat to achieving elimination.**

Authors:

Dr Kathleen Bryce, Institute for Global Health, University College London; Royal Free London NHS Foundation Trust; orcid.org/0000-0001-9348-999X

Dr Colette Smith, Institute for Global Health, University College London

Professor Alison Rodger, Institute for Global Health, University College London; Royal Free London NHS Foundation Trust

Dr Douglas Macdonald, Institute for Liver and Digestive Health, University College London; Royal Free London NHS Foundation Trust

Corresponding author: Douglas Macdonald, Room LG 631, Royal Free Campus, Rowland Hill Street, London. Tel: 02077940500, email: [douglasmacdonald@nhs.net](mailto:douglasmacdonald@nhs.net)

Table of Contents

Methods and Results – page 2

Supplementary Table – page 3

Supplementary Figure – page 4

**Methods and Results**

Patients assessed for treatment in the prison setting (n=79) were excluded from the Cox regression model and a subset time-to-treatment analysis was performed (Supplementary Table). This showed that assessment for treatment in a drug and alcohol service (as compared to in hospital outpatients), being born in the UK, requiring renal dialysis, having hepatitis C genotype other than 1 or 4 and the year of treatment referral persisted in this setting as predictors of a significantly reduced likelihood of starting treatment. Alcohol was now non-significant. HIV remained associated with an increased likelihood of treatment initiation.

To determine whether the average duration of time between approval and treatment is increasing as the programme progresses, this was plotted by month of treatment date (Supplementary Figure). Although this showed a clear significant upward trend, the analysis is confounded by lead-time bias because those treated more recently have a longer potential interval from approval.

**Supplementary Table. Factors associated with progression to HCV treatment uptake - subset analysis in all settings except prison.** Cox regression analysis showing unadjusted and adjusted impact of variables on hepatitis C DAA treatment initiation after approval.

|  | **Unadjusted** | | | **Adjusted** | | |
| --- | --- | --- | --- | --- | --- | --- |
| **Variable** | **HR** | **95% CI** | **p** | **aHR** | **95% CI** | **p** |
| Age (per 10 years) | 0.98 | 0.95, 1.02 | 0.28 | 0.97 | 0.94, 1.01 | 0.09 |
| Male gender (vs. Female) | 0.99 | 0.90, 1.07 | 0.73 | 0.99 | 0.91, 1.09 | 0.90 |
| Pathway setting  Drug and alcohol service  Hospital outpatients | 0.71  REF^†^ | 0.63, 0.80 | <0.001 | 0.81  REF^†^ | 0.70, 0.94 | 0.01 |
| Injection drug use (ever) (Yes vs. No) | 0.78 | 0.72, 0.85 | <0.001 | 0.91 | 0.83, 1.01 | 0.07 |
| Born in UK (Yes vs. No) | 0.85 | 0.78, 0.93 | <0.001 | 0.89 | 0.81, 0.97 | 0.01 |
| Previous treatment (Yes vs. No) | 1.06 | 0.96, 1.17 | 0.28 | 0.99 | 0.89, 1.10 | 0.79 |
| Hepatocellular carcinoma (Yes vs. No) | 0.71 | 0.45, 1.13 | 0.15 | 0.72 | 0.45, 1.15 | 0.16 |
| Alcohol contributing to liver disease (Yes vs. No) | 0.77 | 0.65, 0.90 | 0.001 | 0.85 | 0.72, 1.01 | 0.06 |
| Liver transplant (Yes vs. No) | 1.15 | 0.85, 1.56 | 0.36 | 1.15 | 0.85, 1.57 | 0.37 |
| Renal dialysis (Yes vs. No) | 0.62 | 0.38, 1.01 | 0.05 | 0.59 | 0.36, 0.97 | 0.04 |
| HIV (Positive vs. Negative) | 1.34 | 1.20, 1.50 | <0.001 | 1.23 | 1.09, 1.39 | 0.001 |
| HCV genotype  Genotype 2, 3, 5, 6 or ‘other’  Genotype 1 or 4 | 0.80  REF^†^ | 0.73, 0.87 | <0.001 | 0.84  REF^†^ | 0.77, 0.91 | <0.001 |
| Cirrhosis status  Compensated cirrhosis  Decompensated cirrhosis  Non-cirrhotic | 1.01  0.75  REF^†^ | 0.90, 1.12  0.56, 1.01 | 0.13 | 1.10  0.85  REF^†^ | 0.99, 1.24  0.63, 1.14 | 0.10 |
| Month of DAA programme at referral (per 12 months) | 0.91 | 0.87, 0.94 | <0.001 | 0.95 | 0.91, 0.99 | 0.01 |

†REF = reference value

**Supplementary Figure. Time from multidisciplinary team (MDT) approval to treatment initiation by month of treatment.** The average delay between MDT approval and treatment (+/- 95% CI) by month of treatment is shown, line shows linear regression R^2^= 0.63, p <0.001.


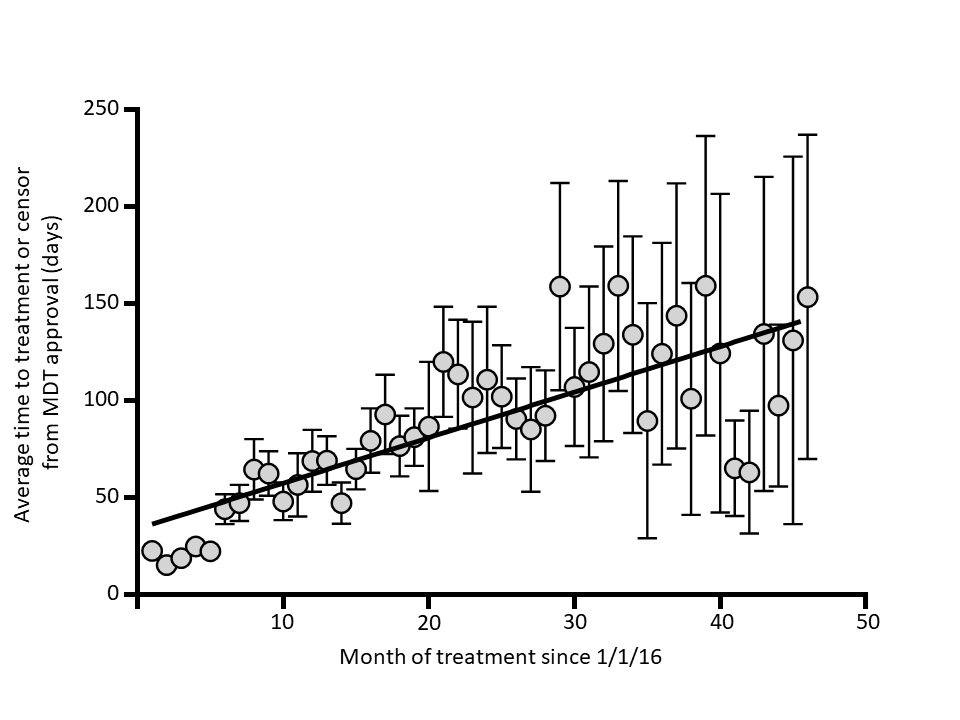

Supplement: Supplementary file 1 — Data S1 [file JVH-30-46-s001.docx]
